# Lipopolysaccharide-induced Notch signaling activation through JNK-dependent pathway regulates inflammatory response

**DOI:** 10.1186/1423-0127-18-56

**Published:** 2011-08-15

**Authors:** Po-Nien Tsao, Shu-Chen Wei, Miao-Tzu Huang, Ming-Cheng Lee, Hung-Chieh Chou, Chien-Yi Chen, Wu-Shiun Hsieh

**Affiliations:** 1 Department of Pediatrics, National Taiwan University Hospital, National Taiwan University College of Medicine, Taipei, Taiwan; 2 Internal Medicine, National Taiwan University Hospital, National Taiwan University College of Medicine, Taipei, Taiwan; 3 Medical Research, National Taiwan University Hospital, National Taiwan University College of Medicine, Taipei, Taiwan

## Abstract

**Background:**

Notch and TLR pathways were found to act cooperatively to activate Notch target genes and to increase the production of TLR-induced cytokines in macrophages. However, the mechanism of LPS-induced Notch activation and its role in sepsis still remains unclear.

**Methods:**

We analyzed the expression patterns of Notch components in a LPS-stimulated murine macrophage cell line using real-time PCR and western blotting. The role of DAPT, a gamma-secretase inhibitor that is known to be a potent Notch inhibitor, in LPS-induced cytokine release and experimental sepsis in mice was also explored. Student's t-test was used to analyze the difference between the two groups.

**Results:**

We found that Notch signaling was activated after LPS stimulation. The expression of Jagged 1, a Notch ligand, induced by LPS occurred in a JNK-dependent manner. In addition, Notch target genes were upregulated by early Notch-independent activation followed by delayed Notch-dependent activation after LPS stimulation. Disruption of Notch signaling by DAPT attenuated the LPS-induced inflammatory responses, including vascular endothelial growth factor (VEGF) and high-mobility group box chromosomal protein 1 (HMGB1), both in vitro and in vivo and partially improved experimental sepsis survival.

**Conclusions:**

These findings support the existence of a synergistic effect of Notch signaling and the LPS pathway both in vitro and in vivo. Therefore, in the future Notch inhibitors may be utilized as adjunctive agents for the treatment of sepsis syndrome.

## Background

Sepsis is a lethal infection-induced systemic inflammatory syndrome and organ dysfunction triggered by bacteria or bacterial products. Sepsis-related mortality is a leading cause of death and is increasing worldwide [1-5]. An overwhelming systemic inflammatory response is the most frequent pathological picture associated with sepsis and leads to fatal multiple organ failure [6,7]. Many basic and clinical studies have focused on targeting proinflammatory mediators implicated in the pathophysiology of sepsis. Unfortunately, most clinical trials so far have not led to an improved overall outcome for persons with this serious medical condition [6-11].

Notch signaling is a highly conserved pathway involved in cell fate decisions, proliferation, and survival [12]. In mammalians, there are four Notch receptors (Notch-1 to -4) and five Notch ligands (Delta-like-1, -3, and -4 and Jagged-1 and -2). Notch-ligand binding leads to the shedding of the Notch extracellular domain and subsequent release of the Notch intracellular domain (NICD) by a γ-secretase complex. The NICD is translocated to the nucleus, where it binds to the transcription factor Rbp-jk and results in the activation of Notch downstream target genes such as basic helix-loop-helix family (Hes1 and Hes5) and hairy and enhancer of split-related (HESR) family (Hey1 and Hey2) [13].

In the immune system, the role of Notch signaling in the development and function of macrophages, NK cells, T cells, B cells, and dendritic cells has been reported [14-18]. Upon infection, Toll-like receptor (TLR) ligands activate macrophages resulting in the production of inflammatory cytokines such as TNF-α, interleukin-1β (IL-1β), and IL-6 [19]. Several Notch receptors and ligands are expressed in both human and mouse macrophages [14,20,21]. Recently, Notch and TLR pathways were found to act cooperatively to activate Notch target genes and to increase the production of TLR-induced cytokines in macrophages [14,22,23]. In addition, some reports also indicated that Notch signaling plays an important role in inflammatory disorders [24,25]. This data allowed us to hypothesize that Notch signaling may play a role in the pathogenesis of sepsis.

Here we report that Notch pathway components are expressed in murine macrophages. LPS-induced Jagged1 (Jag1) was expressed in a JNK-dependent manner. By using loss-of-function and gain-of-function models in vitro, we demonstrate that Notch signaling amplifies the production of LPS-induced inflammatory cytokines including the free form of vascular endothelial growth factor (VEGF) by attenuating the secretion of soluble Flt-1 (sFlt-1). Finally, pharmacological inhibition of Notch activation attenuates the endotoxemia response and partially improves the survival rate of experimental sepsis. We conclude that activation of the Notch pathway in macrophages is important in the development of sepsis and could represent a new adjuvant therapy.

## Materials and methods

### Cell culture and reagents

Murine macrophage-like RAW 264.7 cells were obtained from the American Type Culture Collection (Manassas, VA, USA) and cultured in DMEM (Gibco BRL, Grand Island, NY, USA) supplemented with 10% fetal bovine serum and 4 mM glutamine. Cells were cultured in the presence of LPS (from *Escherichia coli *0111:B4; Sigma-Aldrich, SL, USA) with or without a Notch inhibitor or activator (see below). Specific MAPK inhibitors, PD98059 (Sigma-Aldrich, SL, USA), SB203580 and SP600125 (both from Calbiochem, CA, USA) were used at the concentrations indicated in the figure legends.

### Animals

The animal protocol was approved by the Animal Care and Use Committee of the National Taiwan University Hospital. C56BL/6 strain mice were obtained from the Animal Center of the College of Medicine, National Taiwan University. The animal room was kept on a 12-hour light/dark cycle with temperature and humidity held constant.

### Endotoxemia

Endotoxemia was induced by an i.p. injection of LPS at a dose of 5 μg/g in PBS. We subjected mice to control (vehicle alone) or to *N*- [*N*-(3,5-difluorophenacetyl)-L-alanyl]-(*S*)-phenylglycine-*t*-butyl ester (DAPT) (100 mg/kg, Sigma-Aldrich, SL, USA). Plasma or tissues were collected at indicated times.

### Cecal ligation and puncture (CLP)

We performed CLP as described [26]. Briefly, we anesthetized the mice with pentobarbital (50 mg/kg, i.p.). Under sterile conditions, a 1-2 cm incision was made in the middle abdomen, and the cecum was exposed. We placed a 3.0 silk suture 5.0 mm from the cecal tip away from the ileocecal valve, punctured through twice with a 23-gauge needle, and extruded a small amount of bowel contents (1 mm). We then placed the cecum back and closed the abdominal cavity with a running suture with 3.0 silk. Mice were resuscitated with 1 ml of saline s.c. and placed on a heating pad until they recovered from the anesthetic. We subjected the mice to vehicle and DAPT treatment and monitored them for survival.

### Inhibition or activation of Notch signaling in vitro and in vivo

To inhibit Notch signaling in vitro, RAW 264.7 cells were treated with DMSO (1 μM) or two different γ-secretase inhibitors (GSIs): DAPT (10 μM) or JLK6 (1 μM, Calbiochem, CA, USA). To activate Notch signaling, we added recombinant soluble Jag1, Dll1, or Dll4 (all from R & D Systems, MN, USA) in medium to a final concentration of 1 μg/ml. To block Notch signaling in vivo, DAPT was dissolved in corn oil containing 10% ethanol, and DAPT (100 mg/kg) or vehicle (10 ml/kg) was subcutaneously administered to endotoxemic mice 3 hours before LPS (15 μg/g) injection or to CLP mice 3 hours before and 24 hours after CLP.

### Quantitative real-time PCR

Total RNA from cells and tissues was isolated using Trizol. (Invitrogen, CA, USA) For reverse transcription, 2 μg of total RNA were transcribed using the iScript cDNA synthesis kit (Bio-Rad, Hercules, CA, USA). Real-time RT-PCR was performed using the DNA Engine Opticon 2 detection system (Bio-Rad, CA, USA) and the iQ SYBR Green supermix (Bio-Rad, CA, USA). The threshold cycle (*C_T_*) values were obtained and the relative concentration of RNA for each gene to GAPDH mRNA was determined using the equation: 2^-Δ*C*T^, where Δ*C*T = (*C*TmRNA-*C*TGAPDHmRNA).

### Western blotting

Western blot analyses of MAPK pathways, NICD, and high-mobility group box chromosomal protein 1 (HMGB1) were performed as previously described [27]. 50 μg of total cell lysate was separated on a 10% SDS-polyacrylamide gel and transferred to a BioTrace PVDF membrane (Pall Corporation, East Hills, NY, USA). The blots were then blocked with 5% milk for 1 hour at room temperature and probed with antibodies for Jagged-1 (Santa Cruz Biotechnology Inc, Santa Cruz, CA, USA), phospho (Thr202/Tyr204) and total ERK1/2, phospho (Thr183/Tyr185) and total c-Jun N-terminal kinase (Jnk), phospho (Thr180/Tyr182) and total p38, cleaved Notch1 (all from Cell Signaling Technology Inc., Danvers, MA, USA), HMGB1 (BD Biosciences, NJ, USA), and mouse actin (Sigma-Aldrich, SL, USA) at 4°C overnight. ECL Plus reagents (Amersham Biosciences, Sweden) and appropriate secondary antibodies (Bio-Rad, CA, USA) were used for the detection of western blots. Quantification of bands of the western blots was performed using Image Lab (Bio-Rad Laboratory).

### Measurement of cytokines, VEGF, and sFlt-1 levels

For measurement of LPS-induced cytokines in conditioned medium and plasma, we collected conditional medium and plasma at various times. Concentrations of TNF-α, IL-1β, IL-6, the free form of VEGF, and sFlt-1 in samples were determined using ELISA kits (R & D, MN, USA). All experiments were performed in triplicate, and the data is expressed as mean ± SEM.

### Statistical analysis

All data is expressed as mean ± SEM. Statistical analysis was performed using SPSS 12.0 for Windows (Statistical Package for Social Sciences, Inc., Chicago, IL, USA). The Student's t-test was used to analyze the difference between the study and control groups; p values less than 0.05 were considered statistically significant.

## Results

### Notch pathway components are present in murine macrophages and activated by LPS stimulation

**Figure 1:**
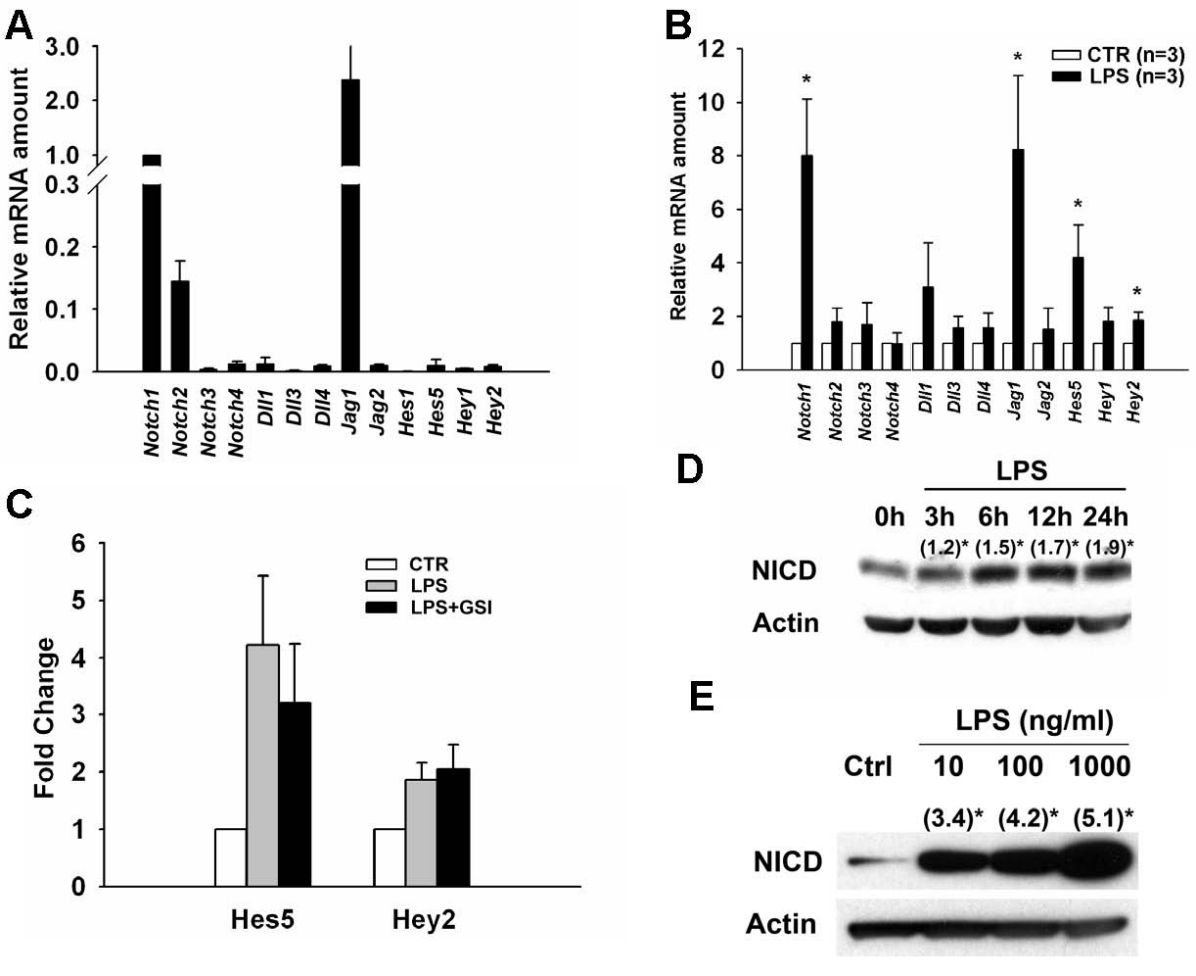
**Expression of Notch pathway components in murine macrophages**. (A) Expression levels of Notch receptors and ligands in RAW 264.7 cells relative to Notch1 expression. Transcript levels were determined by real-time PCR and normalized to GAPDH. (B) Expression levels of Notch receptors and ligands with or without LPS stimulation (1 μg/ml) for 3 hours. Levels of the control group were set as 1. (C) The γ-secretase inhibitor DAPT (10 μM) did not prevent LPS-induced Hes5 and Hey2 expression. (D, E) LPS induced the formation of NICD in both dose- and time-dependent manners. (), fold change as compared to basal level; *, *P *< 0.05.

To identify the Notch pathway components in murine macrophages, we measured their expressions in RAW 264.7 cells using real-time PCR. We found that murine macrophages constitutively expressed almost all Notch receptors and ligands, although the levels of Notch3, Dll3, and Hes1 were very low compared to Notch1 (Figure 1A). The most abundant Notch components expressed in unstimulated RAW 264.7 cells were Notch1, Notch2, and Jag1 (Figure 1A).

Next, we asked whether LPS can activate Notch signaling. We found that the levels of Notch1, Jag1, Hes5, and Hey2 were significantly increased at 3 hours after LPS stimulation as compared to levels in unstimulated cells (Figure 1B). Surprisingly, disruption of Notch signaling by a γ-secretase inhibitor did not prevent this early phase of LPS-induced *Hes5 *and *Hey2 *expression (Figure 1C). In addition, we found that the Notch1 intracellular domain (NICD), the activated form of Notch1, was not induced by LPS at 3 hours after stimulation. However, its expression was increased at 6 hours after LPS stimulation (Figure 1D). The stimulatory effect of LPS (10-1000 ng/ml) on the production of NICD in macrophages was found to be dose-dependent at 24 hours after stimulation (Figure 1E).

### Activation of JNK is required for LPS-induced *Jag1 *expression

**Figure 2:**
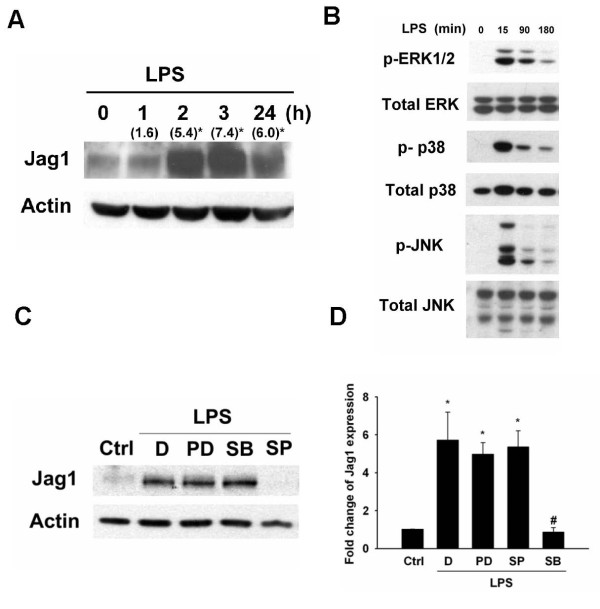
**JNK activation is required for LPS-induced Jag1 expression in macrophages**. (A) Time course of LPS-induced expression of Jag1 protein. (), fold change as compared to basal level (B) MAPK signal pathways were activated by LPS stimulation in macrophages. (C) Cells were pretreated with 10 μM PD98059 (PD), 2 μM SB203580 (SB), 50 μM SP600125 (SP), or DMSO (D) as control 30 minutes before LPS stimulation, respectively. LPS-induced Jag1 expression was completely blocked by SP600125, a JNK inhibitor. (D) Relative levels of Jag1 protein were determined by densitometry (n = 3). The results represent the mean ± SD of three independent experiments. *, *P *< 0.05 compared to control; #, *P *< 0.05 compared to LPS only.

To understand the mechanism underlying the activation of Notch signaling by LPS, we first examined the temporal expression of *Jag1*, as this ligand was robustly induced Notch pathway component by LPS stimulation. We found that *Jag1 *was significantly upregulated (about 4.5-fold) in macrophages at 2 hours after LPS stimulation (Figure 2A). In addition, LPS also activated all three MAPK pathways within 15 minutes (Figure 2B). To investigate whether LPS-induced activation of MAPK pathways is required for LPS-induced *Jag1 *expression, we pretreated cells with specific MAPK inhibitors 30 minutes before LPS stimulation. We found that SP600125 (SP; c-jun NH2-terminal kinase (JNK) inhibitor), but not PD 98059 (PD; ERK inhibitor) or SB203580 (SB; p38 MPAK inhibitor), completely blocked LPS-induced *Jag1 *expression (Figure 2C&D). These results indicate that LPS-induced *Jag1 *expression is JNK-dependent.

### DAPT attenuated the production of LPS-induced cytokines through blocking Notch signaling

**Figure 3:**
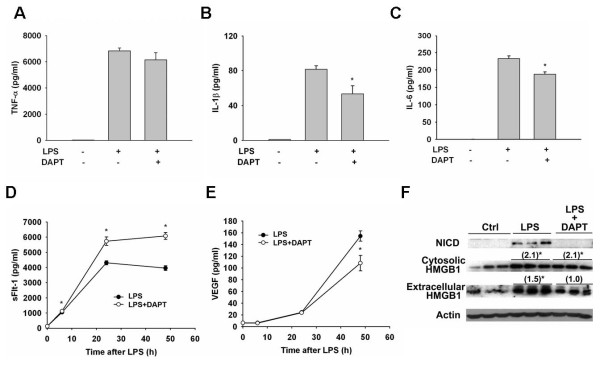
**DAPT inhibited Notch signaling and attenuated LPS-induced inflammatory responses**. DAPT (10 μM) attenuated IL-1β (B), IL-6 (C), but not TNF-α (A) secretion in conditioned medium 6 hours after LPS (1 μg/ml) stimulation. DAPT also amplified the LPS-induced sFlt-1 (D), an anti-inflammatory factor, and attenuated VEGF (E) secretion in a relatively late phase. (F) DAPT, indeed, prevented LPS-induced NICD formation and inhibited HMGB1 secretion, but not production in macrophages 24 hours after LPS stimulation. (), fold change as compared to basal level; *, *P *< 0.05.

To test whether Notch signaling is required for LPS-induced secretion of cytokines in vitro, we investigated the cytokine response in LPS-stimulated mouse macrophage RAW 264.7 cells by inhibiting γ-secretase with DAPT. Treatment of murine macrophages with DAPT did not lead to increased cell death as measured by an MTS assay (data not shown). In the absence of DAPT, the levels of TNF-α, IL-1β, and IL-6 in conditioned medium significantly increased at 6 hours after LPS stimulation. The addition of DAPT partially, but significantly, attenuated the LPS-induced increase in the levels of released IL-1β and IL-6 (35% and 20% reduction, respectively), but did not alter the level of TNF-α (Figure 3A-C). As indicated in our previous report [26], the level of soluble Flt-1 (sFlt-1), an anti-inflammatory factor, in conditioned medium increased progressively between 6 and 48 hours after LPS stimulation. DAPT treatment significantly increased the sFlt-1 levels induced by LPS (Figure 3D). In contrast, the level of the free form of vascular endothelial growth factor (VEGF) significantly decreased upon DAPT pretreatment to 70% of that of the control group at 48 hours after LPS stimulation (Figure 3E). We also measured the LPS-induced secretion of the late mediator, extracellular HMGB1, in conditioned medium using western blotting. Cytosolic and extracellular HMGB1 levels were increased 2- and 1.5-fold, respectively, at 24 hours after LPS stimulation. Interestingly, DAPT pretreatment significantly attenuated the LPS-induced secretion of extracellular HMGB1 into conditioned medium, but did not change the LPS-induced cytosolic HMGB1 expression (Figure 3F).

To test the specificity of the DAPT effect on Notch signaling inhibition, we used JLK6, another γ-secretase inhibitor which cannot prevent NICD formation, instead of DAPT under the same experimental conditions. Our results showed that JLK6 had no effect on LPS-induced cytokine production (data not shown). Taken together, our results indicate that DAPT attenuated the production of LPS-induced cytokines by blocking Notch signaling.

### Activation of notch signaling by soluble notch ligands amplifies cytokine production in LPS-stimulated macrophages

**Figure 4:**
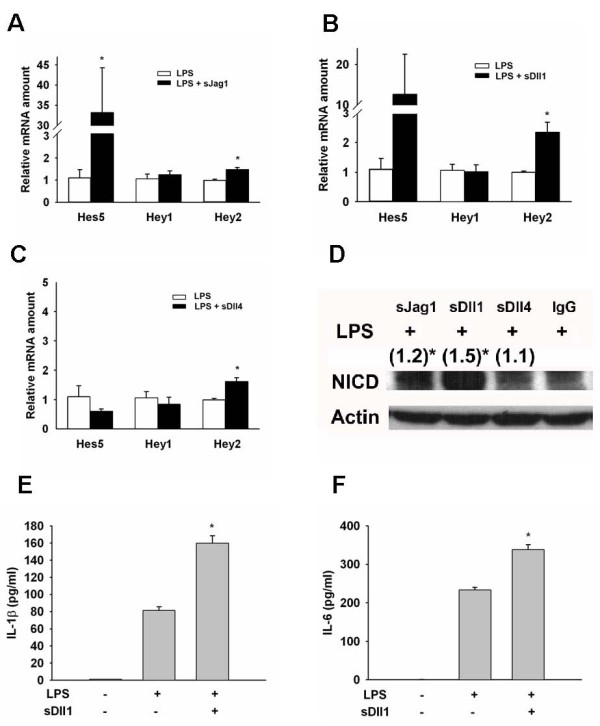
**Soluble Notch ligands activated Notch signaling and amplified LPS-induced inflammatory responses**. (A) Soluble Jagged1 (sJag1; 1 μg/ml) augmented LPS-induced Hes5 and Hey2 mRNA expression in macrophages. sDll1 (B) and sDll4 (C) also had a similar effect. (D) sJag1, sDll1, and sDll4, especially sDll1 (all 1 μg/ml), significantly enhanced LPS-induced NICD formation. (), fold change as compared to basal level. sDll1 significantly amplified LPS-induced IL-1β (E) and IL-6 (F) secretion in macrophages. *, *P *< 0.05.

To further confirm the interplay between Notch signaling and LPS and their synergistic effect on the induction of proinflammatory cytokines in murine macrophages, we measured the levels of IL-1β and IL-6 in conditioned medium at 6 hours after stimulation with LPS with or without the further addition of soluble Notch ligands. By using real-time PCR, we found that treatment with exogenous soluble Jag1, Dll1, or Dll4 resulted in modest upregulation of *Hey2 *expression (Figure 4A-C). Furthermore, Jag1 significantly upregulated *Hes5 *(Figure 4A); the higher mRNA level determined for *Hes5 *at treatment with Dll1 does not appear to be statistically significant (Figure 4B). Interestingly, D114 had no effect on Hes5 (Figure 4C). In addition, the level of NICD was modestly increased in Jag1- or Dll1-treated macrophages (Figure 4D). This data shows that the treatment of murine macrophages with exogenous Notch ligands, especially Jag1 or Dll1, can further engage Notch receptors, resulting in enhanced Notch activation in LPS-treated macrophages.

Next, we measured the levels of IL-1β and IL-6 in conditioned medium using ELISA. We found that soluble Dll1 significantly amplified the LPS-induced secretions of both IL-1β and IL-6 (Figure 4E, F). Soluble Jag1 had a similar effect (data not shown). The effects by Dll1 and Jag1 can be rescued by DAPT (data not shown). However, activating Notch signaling by soluble Dll1 without LPS did not induce IL-1β or IL-6 secretion in murine macrophages. This data further supports the notion that Notch signaling synergizes with the LPS pathway to stimulate the production of proinflammatory cytokines.

### Blocking notch signaling attenuates LPS-induced inflammatory cytokine production in vivo

**Figure 5:**
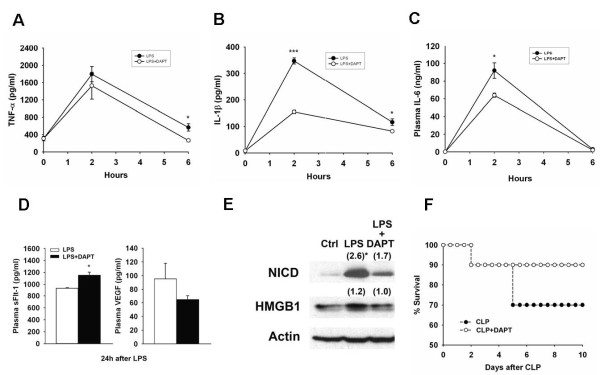
**Protective effect of DAPT against endotoxemia and experimental sepsis**. Pretreatment with DAPT (100 mg/kg) dissolved in corn oil attenuated a sublethal dose of LPS (5 μg/kg) induced TNF-α (A), IL-1β (B), and IL-6 (C) secretion in plasma. (D) LPS-induced sFlt-1 secretion was enhanced by DAPT treatment which resulted in decreased plasma free form VEGF levels. (E) DAPT pretreatment decreased LPS-induced HMGB1 expression in liver tissues. (), fold change as compared to basal level (F) Pretreatment with DAPT partially protected mice from CLP-induced lethality (n = 10 mice per group). *, *P *< 0.05.

To determine the contribution of endogenously activated Notch signaling to inflammatory cytokine production in vivo, we measured the plasma levels of TNF-α, IL-1β, IL-6, sFlt-1, and VEGF in mice injected with LPS (5 mg/kg) with or without DAPT pretreatment. While the peak levels of IL-1β and IL-6 were significantly decreased (55% and 30%) in the DAPT-treated group, the peak levels of TNF-α were only slightly reduced as compared to the LPS-only group (Figure 5A-C). In addition, the plasma sFlt-1 levels significantly increased in the presence of DAPT, resulting in a 33% decrease of free VEGF levels (Figure 5D) in DAPT-/LPS-treated mice. Furthermore, DAPT pretreatment attenuated the LPS- NICD production of NICD and HMGB1 (Figure 5E).

Finally, to study whether blockage of Notch signaling by DAPT protects against lethality in experimental sepsis, we performed LPS-induced endotoxemia and CLP experiments on mice with or without DAPT pretreatment. However, we found that DAPT treatment only partially improved the survival rate of CLP-induced lethality from 70% in the absence of DAPT to 90% with pretreatment (Figure 5F).

## Discussion

The present study supports our hypothesis that the Notch pathway orchestrates LPS signaling in macrophages and experimental sepsis. Our data demonstrates that LPS-induced Notch activation occurs in a dose- and time-dependent manner in macrophages. By using a pharmacological approach to add exogenous soluble ligands to activate Notch signaling, we showed that LPS-induced Notch activation amplifies the inflammatory response to LPS stimulation in macrophages. Finally, we demonstrated a partially protective effect of a Notch signaling inhibitor in experimental sepsis.

Several studies have shown that Notch signaling components exist in both human and murine macrophages [14,20,21,23], although their expression patterns were different among different species. Jag1-Notch1 signaling regulated gene expression in activated macrophages has also been shown [20,21]. Here, we demonstrated that not only Jag1, but also Dll1 can increase the activation of Notch1 and increase the expression of Hes5 and Hey2 induced by LPS stimulation. However, in contrast to data reported with human macrophages [14], we found that Dll4 has no or only a small effect on LPS-induced Notch activation in murine macrophages. This discrepancy may be due to the fact that macrophages of different species were examined. In addition, our data showed that Jag1, Dll1 and Dll4 have differential effects on the expression of Notch target genes after LPS stimulation. Further studies are needed to address this issue.

Recently, Hu et al. showed that LPS stimulation directly induced the expression of the canonical Notch target genes Hes1 and Hey1 without increasing the expression of NICD or Notch ligands within 1-6 hours of stimulation in human primary macrophages [23]. However, the mechanism of induction is still unclear. In this study, we found that the canonical Notch target genes Hes5 and Hey2, as well as Notch receptors and ligands were upregulated within 3 hours after LPS stimulation. However, during the first 3 hours of LPS stimulation, no obvious increase in the expression of NICD was found. In addition, disruption of Notch signaling by the γ-secretase inhibitor DAPT did not prevent LPS-induced Hes5 and Hey2 expression at 3 hours after LPS stimulation. This data suggests that LPS-induced expression of Notch target genes consists of an immediate-early Notch-independent activation and is followed by a delayed, indirect Notch-dependent activation.

Strikingly, Jag1 was rapidly induced by LPS stimulation in murine macrophages. By using a pharmacological approach, we clearly demonstrated that LPS-induced Jag1 expression is mediated by a JNK-dependent pathway. This finding is in agreement with recent results demonstrating that Jag1 is a direct target of TLR signaling and that the induction of Jag1 expression is partially dependent on canonical TLR-activated NF-kB and MAPK signaling pathways [22].

What is the role of Notch signaling in LPS-induced inflammatory responses? We demonstrated that DAPT significantly attenuated the secretion of intermediate (IL-1β and IL-6) and late inflammatory cytokines (VEGF and HMGB1), but not of an early inflammatory cytokine (TNF-α). This result is reasonable because DAPT can only prevent the cytokine response at 3 hours after LPS-induced Notch activation. However, the effect of Notch inhibition on LPS-induced proinflammatory cytokines was modest. In contrast, JNK6, a GSI that does not influence Notch activity, has no anti-inflammatory effect. Furthermore, the activation of Notch signaling by exogenous Notch ligands has the opposite effect on the production of LPS-induced proinflammatory cytokines. Together with this data, this suggests that DAPT specifically inhibits LPS-induced Notch activation followed by attenuating the inflammatory responses.

Another interesting finding is that DAPT increased sFlt-1 secretion and decreased VEGF secretion in LPS-treated macrophages. Previously, we and others reported the protective effect of sFlt-1 through inhibiting VEGF signaling in experimental sepsis [26,28]. In this study, we observed a continuous increase in sFlt-1 secretion from 6 to 48 hours after LPS stimulation. By contrast, VEGF secretion induced by LPS was attenuated until 48 hours after LPS stimulation. This data suggests that the reduced levels of the free form of VEGF, an inflammatory cytokine, was a consequence of the increased levels of sFlt-1 as its binding to VEGF decreases the free form of VEGF [29]. To the best of our knowledge, our study is the first report demonstrating that a disruption of LPS-induced Notch activation can amplify the effect of LPS-induced sFlt-1 secretion and modulate VEGF activity.

Finally, the protective effect of DAPT treatment in vivo is moderate in this study. There are some possibilities to explain this result: (1) the dose of DAPT or the timing of treatment may not be optimal considering the relatively short half-life of DAPT [30]; (2) DAPT only has a partial effect on attenuating LPS-induced inflammatory cytokines secretion as shown in our in vitro experiments; (3) the Notch signaling network is more complicated in vivo than in vitro, so a GSI only has a partially protective effect in vivo. However, using a genetic approach, Hu *et al. *demonstrated that deletion of RBP-J in the myeloid compartment protected mice from endotoxin lethality in vivo [23]. In addition, we also showed that blocking Notch signaling by DAPT injection attenuated LPS-induced inflammatory cytokine production in vivo (Figure 5). This data suggests that modulation of Notch signaling by a γ-secretase inhibitor might be an adjuvant pharmacologic strategy to improve the treatment of sepsis.

## Conclusions

In summary, we demonstrated that Notch target genes can be induced by LPS in both Notch-independent and Notch-dependent manners. The LPS-induced intermediate and late inflammatory responses were attenuated by the γ-secretase inhibitor DAPT and amplified by soluble Notch ligands in vitro. Furthermore, we demonstrated that DAPT has a partially protective effect on experimental sepsis in vivo. Our data therefore suggest that a Notch inhibitor holds potential as an adjunctive agent for the treatment of sepsis syndrome.

## List of abbreviations

NICD: Notch intracellular domain; TLR: Toll-like receptor; VEGF: vascular endothelial growth factor; sFlt-1: soluble Flt-1; CLP: cecal ligation and puncture; DAPT: *N*- [*N*-(3,5-difluorophenacetyl)-L-alanyl]-(*S*)-phenylglycine-*t*-butyl ester.

## Competing interests

The authors declare that they have no competing interests.

## Authors' contributions

PNT designed this study, interpreted the data, and drafted the manuscript. SCW and MTH participated in the design and coordination of this study, and performed experiments. MCL performed the experiments pertaining to the MAPK pathway. HCC, CYC and WSH participated in the analysis and interpretation of data. All authors read and approved the final manuscript.
